# Choroid plexus organoids mimic amyloid uptake at the blood-cerebrospinal fluid-barrier

**DOI:** 10.3389/fncel.2026.1769911

**Published:** 2026-02-18

**Authors:** C. Municio, K. Sapidou, E. J. Apsley, M. Fernandez-Otero, C. E. Arber, S. Wray, E. Carro, L. Pellegrini

**Affiliations:** 1 Neurobiology of Alzheimer’s Disease Unit, Functional Unit for Research into Chronic Diseases, Network Centre for Biomedical Research in Neurodegenerative Diseases (CIBERNED), Instituto de Salud Carlos III, Madrid, Spain; 2 Centre for Developmental Neurobiology, King’s College London, London, United Kingdom; 3 Department of Neurodegenerative Disease, Queen Square Institute of Neurology, University College London, London, United Kingdom

**Keywords:** Alzheimer’s disease, amyloid-beta, blood-cerebrospinal fluid-barrier (B-CSF-B), choroid plexus (ChP), organoid

## Abstract

The choroid plexus (ChP) is a specialised tissue of the central nervous system that produces cerebrospinal fluid (CSF), maintains cerebral homeostasis and forms the blood-CSF barrier (B-CSF-B), a key interface that regulates the exchange of substances between the blood and the brain. Despite its physiological importance, the involvement of the ChP in neurodegenerative diseases such as Alzheimer’s disease (AD), remains poorly understood. This is largely due to the reliance on murine models and the limited availability of human brain tissue. Recent advances in human stem-cell derived ChP organoids now offer a more physiologically relevant model to interrogate ChP role in human health and disease. Given that in AD pathology beta-amyloid (Aβ) accumulation has been linked to early disruption of brain barriers, studying the B-CSF-B is particularly relevant. Transthyretin (TTR), the predominant protein secreted by the ChP, is thought to play a role in the transport and clearance of Aβ, although its exact mechanisms are not yet fully elucidated. Here, we propose the use of ChP organoids to investigate the role of the B-CSF-B in amyloid uptake which may contribute to barrier dysfunction and disease progression in AD.

## 1 Introduction

The choroid plexus (ChP) is a highly specialised structure located within all four ventricles in the central nervous system (CNS). Its primary function is the production and regulation of cerebrospinal fluid (CSF), with an adult human producing around 500 mL of CSF daily, maintaining a circulating volume of approximately 150 mL (Lun et al., 2015). The ChP also forms the blood-CSF barrier (B-CSF-B), a selective interface that tightly regulates which substances enter the brain from the bloodstream (Ghersi-Egea et al., 2018; Saunders et al., 2023). The continuous flow and turnover of CSF are crucial for brain homeostasis, nutrient and signal distribution, facilitating the bulk removal of waste products and metabolites through the glymphatic system (Municio et al., 2023; Keil et al., 2025). Beyond its barrier and secretory functions, the ChP plays vital roles in maintaining overall brain homeostasis, participating in immune surveillance and facilitating the distribution of essential hormones and neurotrophic factors (Lun et al., 2015; Bitanihirwe et al., 2022).

Structurally, the ChP is a sheet-like, villous structure composed of a polarised epithelial cell monolayer surrounding a core of highly vascularised connective tissue, which contains stromal cells, immune cells and fenestrated capillaries (Dani et al., 2021). These fenestrations allow for rapid filtration of plasma into the interstitial space of the ChP by hydrostatic pressure (Bitanihirwe et al., 2022). ChP epithelial cells exhibit a high concentration of tight junction proteins as well as cell adhesion molecules. These junctional complexes seal the paracellular space, thereby limiting the free diffusion of most substances and enabling the ChP to strictly control the composition of the CSF with precision (Liu et al., 2022). Beyond its secretory and barrier roles, the ChP also secretes hormones and neurotrophic factors and contributes to CNS immune regulation (Saunders et al., 2023; Santos et al., 2017; Xu et al., 2025).

Alterations in the ChP structure are associated with various neurodegenerative conditions, including AD (Liu et al., 2022; Sharma et al., 2025), which is currently the most common cause of dementia and is diagnosed by measuring Aβ in CSF or brain (Guo et al., 2020). In AD, the ChP undergoes significant structural and functional impairments. Pathological changes include cellular morphological alterations, such as epithelial cell atrophy and flattening, along with a thickening and irregularity of the basement membrane (Mesquita et al., 2012). Critically, there is a decrease in tight junction proteins, leading to increased membrane permeability and potential alterations in leukocyte passage into the CSF (Liu et al., 2022; Gião et al., 2022). Mitochondrial dysfunction, oxidative stress and the presence of Aβ deposits have also been reported in the epithelial cells of the ChP (Delvenne et al., 2024). Some of these abnormalities may be difficult to distinguish from age-related changes, such as volume loss, fibrosis or toxin accumulation (Mesquita et al., 2012).

AD is also associated with decreased CSF production and impaired flow, which consequently reduces the secretion of essential neuroprotective proteins and impacts the clearance of neurotoxic molecules like Aβ (Attier-Zmudka et al., 2019; Kant et al., 2018; Farinas et al., 2025; de Leon et al., 2017). Amyloid uptake and clearance is carried out by specific carriers, including: ATP-binding cassette transporters; receptor for advanced glycation end products (RAGE); low-density lipoprotein receptor related protein 1 (LRP1) and megalin (LRP2); apolipoprotein J (APOJ) and transthyretin (TTR) (Pascale et al., 2011; Crossgrove et al., 2005; González-Marrero et al., 2015; Deane et al., 2004). TTR, the major protein secreted by the ChP, can bind Aβ, preventing its aggregation and toxicity and has also been reported to facilitate Aβ fibril disruption (Duarte et al., 2016).

Historically, technical difficulties and limitations have hindered the comprehensive study of the human ChP. The emergence of human ChP organoids now enables the study of the B-CSF-B in a model that recapitulates multiple cell types of the ChP, secretory CSF function and developmental timing. These advanced models offer unprecedented opportunities to investigate early disease-relevant mechanisms such as impaired barrier integrity, altered CSF secretion and to dissect the complex interplay between the ChP cell types (Pellegrini et al., 2020; Pellegrini and Lancaster, 2021; Tang et al., 2025; Masters et al., 2025).

In the present study, we evaluated the robustness of the human ChP organoid model as a tool for studying the role of the ChP in amyloid uptake. First, we show increasing expression levels and localisation of relevant amyloid transporters directly linked to AD in the ChP organoid model over time. Second, we analysed the effect of Aβ on the expression of these parameters as well as amyloid binding protein TTR. Together, our preliminary findings show that this model can fill important knowledge gaps in ChP biology and provide a controlled framework for investigating the development and progression of AD in a human model that recapitulates the complex cellular environment and function of the ChP.

## 2 Materials and methods

### 2.1 Stem cell culture and ChP organoid culture conditions

Human embryonic stem (hES) cells H1 and H9 (WiCell WA01, WA09) were approved for use by the Medicines and Healthcare products Regulatory Agency (Reference #SCSC23-57). Cells were cultured in 6-well plates coated in Matrigel Growth Factor reduced Basement Membrane Matrix (Corning, 356,230) and maintained in StemFlex medium (Gibco, A3349401). STEMdiff Choroid Plexus Organoid Differentiation Kit (STEMCELL Tech., 100–0824) was used to generate ChP organoids following kit instructions. Organoids were collected at different time points (batch breakdowns in Supplementary Table 1). Where applicable, the organoids were incubated in the presence of 1 μg/mL β-Amyloid (1–42), HiLyte Fluor™ 488-labeled (AS-60479-01, AnaSpec) or scrambled Amyloid peptide as a negative control from 2 days to 1 week and snap-frozen in liquid nitrogen.

### 2.2 RNA isolation and RT-PCR

Three separate batches with three organoids per batch were used for each condition. Total RNA was isolated from ChP organoids using the Monarch® Spin RNA Isolation Kit (New England Biolabs, # T2110S), following the manufacturer’s recommended protocol. The RNA concentration was measured using a NanoDrop™ One Spectrophotometer (Thermo Fisher Scientific), and 1 μg of each sample was retrotranscribed into cDNA using an iScript™ cDNA Synthesis Kit (Bio-Rad Laboratories). Quantitative real-time PCR (qRT-PCR) was performed using a LightCycler® 480 Instrument (Roche Diagnostics) and NZYSpeedy qPCR Green Master Mix (NZYTech). Predesigned primers were used in the qRT-PCR to determine the expression levels of TTR (CTGGAAGGCACTTGGCATCT and GACAGCCGTGGTGGAATAGG), LRP1 (CTGGCGAACAAACACACTGG and CACGGTCCGGTTGTAGTTGA) and Actin (GCCGCCAGCTCACCATGGATG and CCATCACGCCCTGGTGCCTGG). Relative mRNA levels were calculated using crossing-point (Cp) data and the ΔΔCp method. Cp data from the gene of interest (GOI) were normalized to the mean of endogenous gene Actin data to obtain ΔCp data (ΔCp = mean CpActin−CpGOI). ΔΔCp was then calculated by comparing the normalized ΔCp values from each time point.

### 2.3 Immunoblotting

Snap-frozen organoids were homogenised in RIPA buffer. Samples containing 10 μg of protein were prepared using NuPAGE LDS Sample Buffer 4x and DTT 1M. Protein samples were loaded into a polyacrylamide gel and transferred to a PVDF membrane (Immobilon) for 3 hours at 4 °C. Membranes were blocked in 5% milk in PBS-T and incubated with the following primary antibodies overnight at 4 °C: sheep anti-TTR (1:1000, Abcam #ab9015), rabbit anti-LRP1 (1:1000, Abcam, #ab92544) and mouse anti-GAPDH (1:1000, Abcam, ab8245), rabbit anti-β-actin (1:1000, Abcam, #ab8227). Secondary antibodies conjugated to Alexa Fluor were added for 1 hour at room temperature (1:500). Membranes were imaged using a Li-COR Odyssey CLx Infrared Imaging System. Densitometric quantification was performed with Image Studio Lite 5.0 software (Li-COR Biosciences, Lincoln, NE, USA). Protein bands were normalized with β-actin or GAPDH levels, as a control loading protein, and the measurements were expressed as a percentage of the control group.

### 2.4 Immunostaining

Organoids were fixed in 4% PFA at 4 °C overnight, then transferred to a 30% sucrose buffer solution at 4 °C for at least 24 h. Organoids were next embedded in a gelatin solution. Frozen blocks were sectioned as previously described (Lancaster and Knoblich, 2014). After blocking and permeabilization (1% NDS, 0.25% Triton-X-100, PBS), sections were incubated at 4 °C overnight with the following primary antibodies: sheep anti-TTR (1:500, Abcam, ab9015), mouse anti-TTR (1:500, Abcam, ab204997), mouse anti-Decorin (DCN) (1:300, Sigma, WH0001634M1), rabbit anti-LRP1 (1:1000, Abcam, #ab92544). Secondary antibodies labelled with Alexa Fluor 488, 568, 647 were applied for 1 hour at room temperature (1:1000). Slides were washed again three times before mounting coverslips with a mounting medium with DAPI (Prolong Diamond, ThermoFisher). Control sections without primary antibody treatment were processed simultaneously. Images were acquired using a Zeiss LSM 780 confocal microscope (Carl Zeiss) and prepared using Fiji (NIH). Intensity of TTR and LRP1 analysis was performed with size filtering and circularity constraints to detect individual positive cells, each detected cell was stored as a region of interest (ROI). The ROIs were chosen using an independent field selection process with inclusion and exclusion rules: positive staining for ChP epithelial markers (TTR and AQP1) and presence of cuboidal monolayers. Each positive cell was compared against DAPI mask. The images were processed in ImageJ software. Immunostaining quantification was measured as mean fluorescence intensity (MFI) from multiple regions (3–4) per organoid (2–3 organoids per batch, 3 batches).

### 2.5 CSF extraction and ELISA

Day 42 organoids were incubated w/wo 1 μg/mL β-Amyloid (1–42) for 3 days. Four batches of H1 derived organoids and one batch of H9 derived organoids (three organoids each pooled) from day 46 to day 132 were used to assess control levels of β-Amyloid across developmental time and grouped as above or below 100 days. Culture medium was retrieved, and CSF was extracted for analysis of Aβ40 and Aβ42 using electrochemiluminescence assays: V-PLEX Human Aβ42 Peptide (6E10) Kit (Meso Scale Discovery) and measured on a Meso QuickPlex SQ 120 according to the manufacturer instructions.

### 2.6 Analysis of single cell RNA seq (scRNA seq)

For scRNAseq analysis, the open data base (NCBI GEO Series: GSE150903) (Pellegrini et al., 2020; Speir et al., 2021) and UCSC cell browser were used. The samples available in the database were two organoids of each condition: 55-day H9 telencephalic organoids, 27-day H1 ChP, 46-day H1 ChP and 53-day H1 ChP. UMAP and Dotplots plots were created using Seurat in RStudio.

### 2.7 Statistical analysis

Statistical analysis and graphs were performed using GraphPad Prism Software version 8.0 (La Jolla, CA, USA). To account for inherent variability in organoid experiments, we treated each independent differentiation/generation as a distinct batch and reported, for every experiment, the number of batches and organoids analysed. Unless otherwise stated, *n* denotes the number of independent generation batches (biological replicates at the level of organoid production), while individual organoids within a batch were considered technical replicates. True biological replication was achieved through the use of independent hESC lines; in this study we used H1 and H9, which were handled interchangeably and have been extensively characterised using the same methodology (Pellegrini et al., 2020). One way ANOVA was used to compare means across different conditions followed by Tuckey’s multiple comparisons test. All the data are representing as the means ± SDs Statistical significance was set up at *p* < 0.05. *p < 0.05; **p < 0.01; **p < 0.001;****p < 0.0001.

## 3 Results

### 3.1 Increased expression of LRP1 over time in human ChP organoids

**Figure 1:**
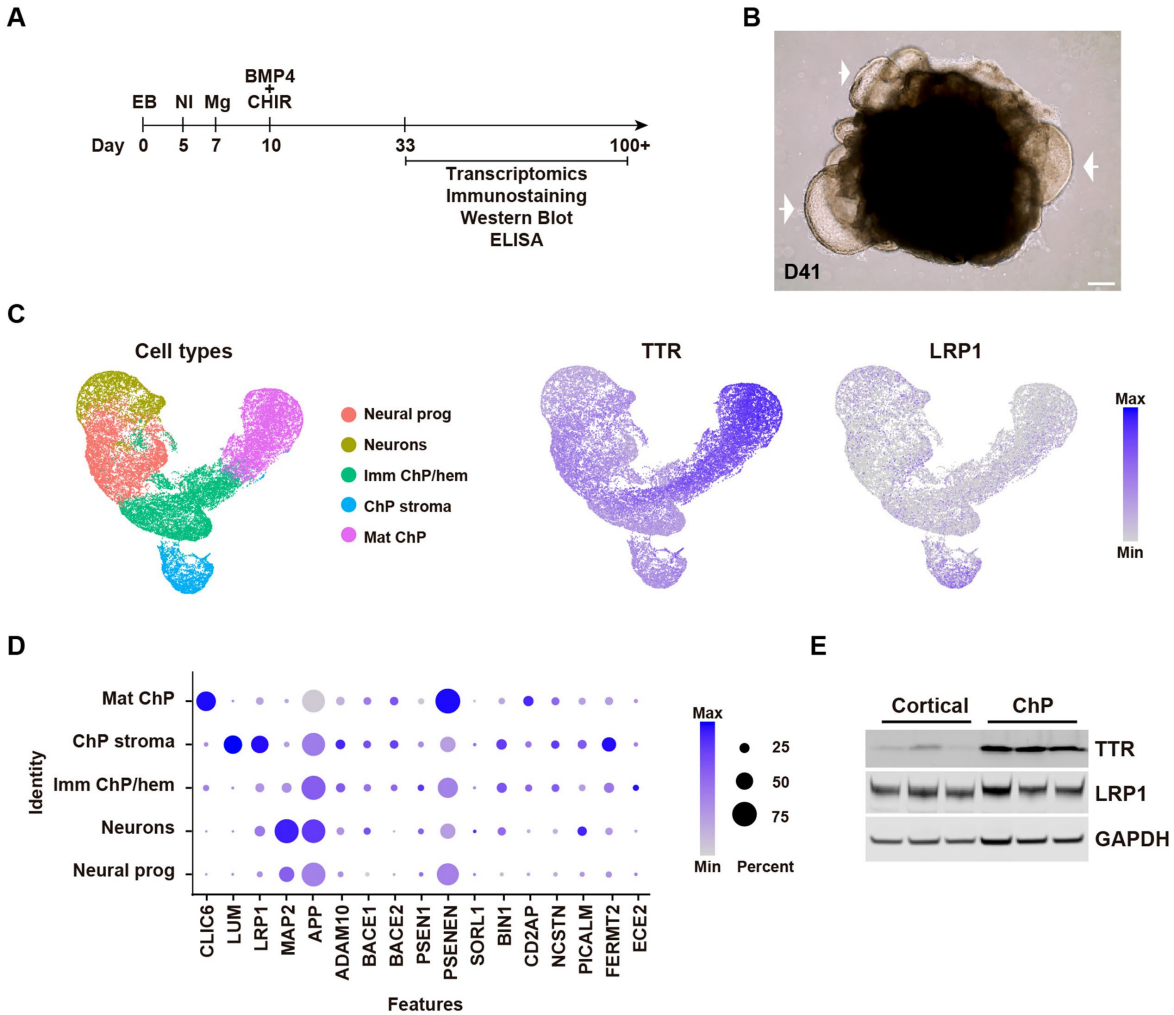
Expression of AD-related genes in human ChP organoids. **(A)** Protocol timeline of ChP organoids over time. **(B)** Representative bright field image of day 41 H1 ChP organoid. Arrow indicates ChP epithelium. Scale bar: 200 μm. **(C)** UMAP plots of the combined samples (one telencephalic H9 organoid and three H1 ChP organoids) showing scRNA-seq clusters of cell types and enrichment of TTR and LRP1 expression. **(D)** Dotplots showing gene expression levels of relevant ChP markers (CLIC6, epithelial cells; LUM, stromal cells and relevant genes involved in APP processing and uptake) from the scRNA-seq data. **(E)** Immunoblot for TTR, LRP1, and the loading control GAPDH of H9 cortical and H9 ChP organoids lysates. Each lane with *n* = 3 independent batches of days 75, 73, and 68 H9 organoids.

To generate human ChP organoids, we used previously established protocol based on the use of the dorsalising factor Bmp4 in combination with the Wnt-activator molecule CHIR (Pellegrini et al., 2020) (Figures 1A,B). To investigate *TTR* and *LRP1* expression in the ChP organoids, we analysed our previously published cell RNA-sequencing (scRNA-seq) database (GSE150903) (Pellegrini et al., 2020; Speir et al., 2021). Combined analysis of one telencephalic organoid and three ChP organoids at various stages of maturation showed largely distinct clusters (Figure 1C). Differential gene expression and cell-specific marker analysis revealed distinct identities of the ChP organoids including immature ChP/hem, mature ChP epithelium, ChP stroma and cortical neurons (Figure 1C). Notably, *TTR* gene expression was predominant in the mature ChP cluster. In contrast, LRP1 expression was distributed among all ChP clusters and was also present in the neuronal and in stromal cell population (Figure 1C). Furthermore, scRNA-seq data revealed the expression levels of relevant ChP markers involved in APP processing (Figure 1D). TTR and LRP1 protein levels were confirmed by Western blot of organoid lysates (Figure 1E), showing, as expected, higher levels of TTR in ChP organoids compared to cortical organoids. LRP1 levels were similar in ChP and cortical organoids (Figure 1E).

**Figure 2:**
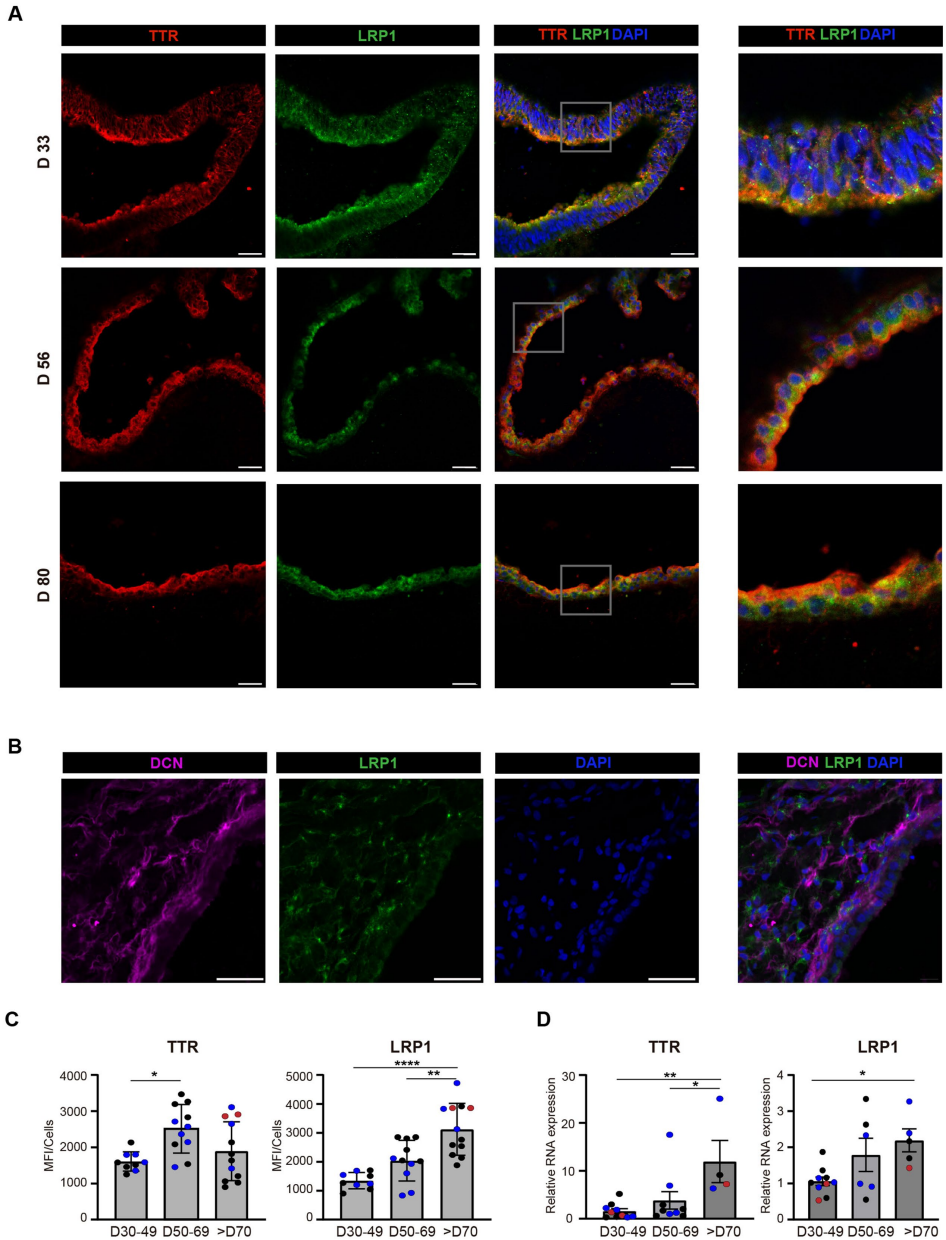
TTR and LRP1 expression in human ChP organoids. **(A)** Immunostaining of different epithelial markers: TTR (red), LRP1 (green), and DAPI (blue) of ChP organoids (day 33 H9; day 56 H9; day 80 H1). Scale bar: 40 μm. **(B)** Immunostaining of stromal marker of H1 ChP organoids (day 56): DCN (magenta), LRP1 (green), and DAPI (blue). Scale bar: 40 μm. **(C)** Immunostaining quantification of TTR and LRP1 presented as mean fluorescent intensity from multiple regions within the organoids (*n* = 2–3 batches of H1/H9 with 2–3 ChP organoids per batch: 30–49 days, 2 batches; 50–69 days, 2 batches; >70 days, 3 batches). Batches are colour-coded: data point colour (blue, red, and black) denotes organoids within the same batch. Data are expressed as mean ± SD. *p < 0.05; **p < 0.01; ****p < 0.0001, using One-way ANOVA and *post hoc* Tukey test (TTR: D30-49 vs. D50-69 *p* = 0.012; LRP1: D30-49 vs. D70 *p* = 0.0001; D50-69 vs. D70 *p* = 0.0026). **(D)** Relative RNA expression of *TTR* and *LRP1* genes in 3 batches of H1 ChP organoids between 30–49 days (10 organoids), 50–69 days (6 organoids) and older than 70 days (5 organoids). Batches are colour-coded: data point colour (blue, red and black) denotes organoids within the same batch. Data are expressed as mean ± SD. *p < 0.05; **p < 0.01; using ne-way ANOVA and post hoc Tukey test (*TTR*: D30-49 vs. D70 *p* = 0.0059; D50-69 vs. D70 *p* = 0.0348; *LRP1*: D30-49 vs. D70 *p* = 0.029). MFI = mean fluorescence intensity.

To examine expression levels of LRP1 over time in the human ChP we performed a time course analysis of ChP organoids grouped according to their maturation age: young (30–49 days), mature (50–69 days) and old (over 70 days). Using confocal microscopy, we confirmed LRP1 localisation in the epithelia and stromal cells of ChP organoids (Figures 2A,B). Fluorescence quantification analyses showed that both TTR and LRP1 levels increased with organoid maturation, showing higher expression levels in the older organoid group (Figure 2C).

PCR analyses performed using whole organoids confirmed that *TTR* gene expression increased with organoid maturation, particularly in the group over 70 days of age. We observed a similar trend for *LRP1*: the expression significantly increases between 49 and 50 days organoids to the older group (over 70 days) (Figure 2D).

### 3.2 Increase in LRP1 in ChP organoids exposed to Aβ42 seeds

**Figure 3:**
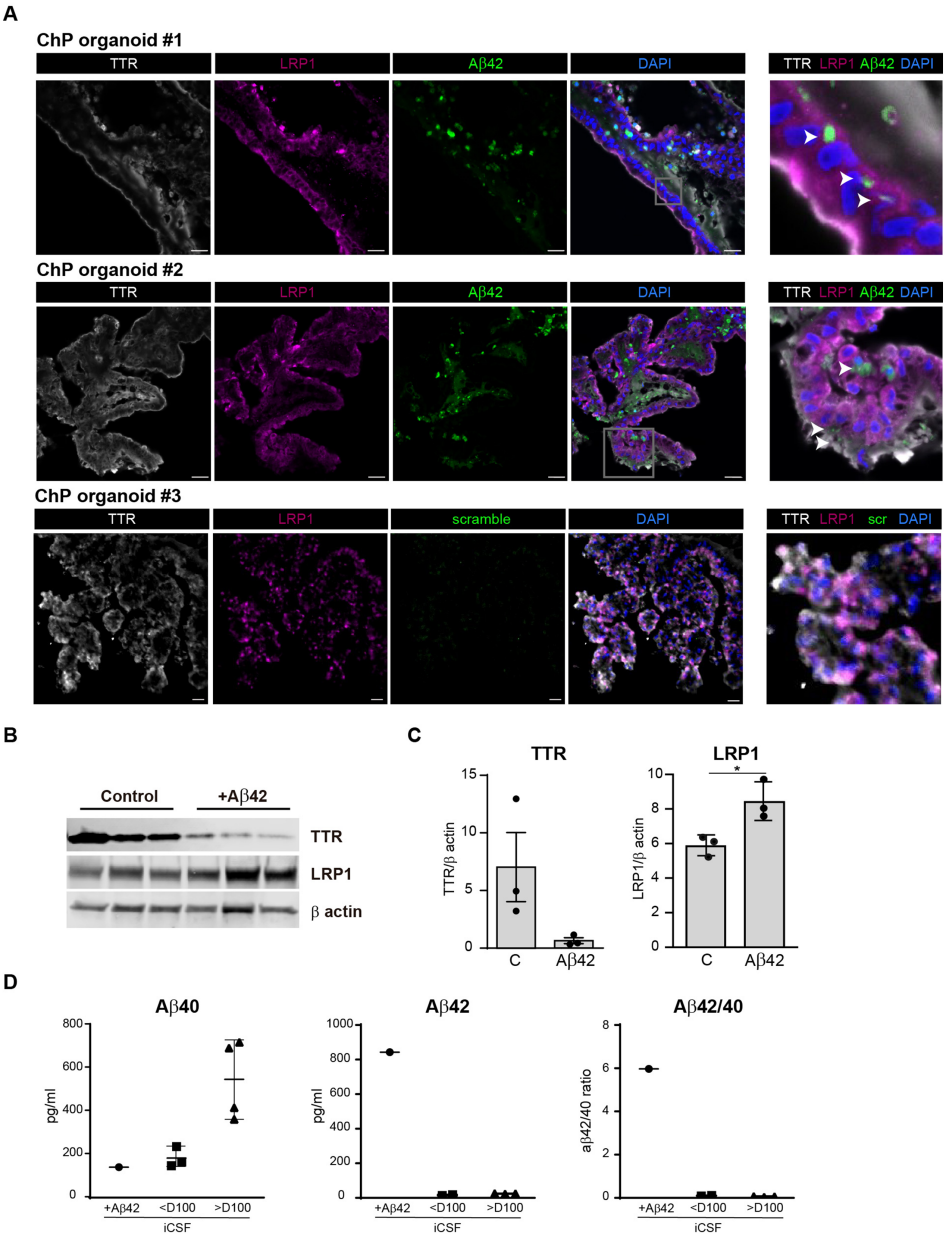
Amyloid uptake in ChP organoids epithelial cells and detection in iCSF. **(A)** Representative confocal images of three H9 ChP organoids (day 49) exposed to 1 μg/mL Aβ42-488 (green) seeds or scramble sequence for 1 week, collected at day 56 and stained for TTR (grey), LRP1 (magenta) and nuclei (DAPI, blue). Arrowhead points to Aβ42 seeds. Scale bar: 20 μm. **(B)** Immunoblots from day 42 H1 ChP organoids cultured for 2 days with 1 μg/mL Aβ42 seeds, probed for TTR, LRP1, and loading control β actin. **(C)** Quantification of immunoblots, band intensity normalized for β actin. Each lane with *n* = 3 independent batches of d42 H1 organoids. Data are expressed as mean ± SD. *p < 0.05, using Mann–Whitney test (LRP1: C *vs* Aβ42 *p* = 0.025). **(D)** Scatter plots show Aβ40 and Aβ42 concentrations in iCSF derived from organoids incubated w/wo 1 μg/mL β-Amyloid (Aβ1-42-488) for 3 days by electrochemiluminescence assays. +Aβ42 represents iCSF from organoid treated with recombinant Aβ42-488 for 3 days (+Aβ42, 1 H1 organoid day 42; <D100, 4 H1/H9 organoids; >D100 3 H1 organoids). Data are expressed as mean ± SD. *p < 0.05, using One-way ANOVA post hoc Tukey test. iCSF = *in vitro* CSF-like.

To assess whether ChP organoids can recapitulate amyloid uptake and seeding, organoids were exposed to fluorescently labelled Aβ42 seeds for 1 week (Figure 3A). Confocal imaging revealed that Aβ42 particles were able to cross the cell membrane and enter the cell cytoplasm, suggesting that epithelial cells can recapitulate amyloid uptake from the basolateral side (Figure 3A).
To assess TTR and LRP1 receptor modulation in response to amyloid exposure, we incubated ChP organoids with Aβ42 seeds for 2 days. A western blot analysis of organoid lysates revealed a decreasing trend in TTR levels in the presence of Aβ42 seeds compared to the control (Figures 3B,C). Conversely, LRP1 levels significantly increased in the presence of the seeds (Figures 3B,C).

Finally, to assess endogenous Aβ isoform levels in organoid *in vitro* CSF-like (iCSF) we cultured organoids at various stages of maturation up to 132 days. The CSF-like fluid was collected and analysed using MSD ELISAs to detect Aβ40 and Aβ42. Organoids older than 100 days produced more Aβ40 than younger ones (Figure 3D). To assess amyloid ability to cross the B-CSF-B, we collected CSF from organoid exposed to Aβ42 seeds for 3 days. As expected, control, unstimulated ChP organoids did not secrete Aβ42 into the CSF-like fluid, whereas Aβ42 was detected in the CSF in the group exposed to the seeds suggesting that ChP organoids recapitulate amyloid uptake and crossing of the B-CSF-B (Figure 3D).

## 4 Discussion

In AD, Aβ accumulates in the brain in the form of amyloid plaques impairing physiological neuronal processes. These Aβ deposits can be caused by overproduction, inadequate metabolic clearance or inappropriate transport across the brain barriers, including the B-CSF-B (Crossgrove et al., 2005; Silverberg et al., 2003). However, there is little evidence regarding the underlying processes of this disruption to the barrier. Some studies suggest that the ChP plays a significant role in the clearance of Aβ42, helping to regulate its levels in the brain (Crossgrove et al., 2005; Alvira-Botero and Carro, 2010). In addition, amyloid-like inclusions have been detected in the ChP during ageing and have been shown to affect ChP morphology and functions. The most notable of these are decreased CSF production and turnover, altered metabolic activity and reduced clearance of Aβ peptides (González-Marrero et al., 2015).

This study represents a proof of principle that organoids can be used to investigate amyloid uptake and early AD-linked pathogenic alterations in the ChP. This organoid model allows us to analyse the barrier mechanisms involved in the transport and clearance of Aβ in greater detail than is possible with 2D cultures, while being more accessible and tractable than animal models. The preliminary data presented in this Brief Report demonstrate that ChP organoids can serve as a sufficiently complex model for studying the molecular mechanisms involved in uptake, barrier crossing and LRP1 receptor modulation.

Here, we confirmed the presence of molecules relevant to the transport and clearance of Aβ, such as TTR and LRP1, using different approaches, including RNA expression analysis, microscopy imaging and protein quantification. In all cases, we detected similar amounts to those previously described in the human ChP, in the same spatial location, which is essential for proper function. To confirm that these proteins were modified in the context of AD, as previously described (Duarte et al., 2016) we exposed the organoids to an environment containing Aβ and assessed the resulting changes. Our preliminary results strongly suggest that the epithelium of ChP organoids can internalise Aβ peptides as previously reported in other 2D *in vitro* ChP models (Masters et al., 2025).

These findings are consistent with previous studies describing Aβ accumulation in the ChP of AD patients and mice models (González-Marrero et al., 2015). Aβ peptide accumulation in the ChP is largely responsible for toxicity and B-CSF-B dysfunction (Vargas et al., 2010). Furthermore, this process results in the impairment of certain Aβ carriers. TTR is one of the main proteins secreted into the CSF that can bind to Aβ and prevent its aggregation. TTR is found in lower concentrations in the CSF of patients with AD (Duarte et al., 2016; Castaño et al., 2006; Gloeckner et al., 2008). Reduced TTR expression has been also reported in APP/PS1 mice (Carro et al., 2002). Consistent with this, ChP organoids exposed to Aβ peptides exhibited a significant decrease in TTR levels. Another potential explanation for TTR reduction could be its aggregation with Aβ resulting in insoluble form. Therefore, the present findings using ChP organoids demonstrate that accumulation of Aβ in ChP might be directly involved in the impaired Aβ clearance in AD.

Most Aβ carriers share LRP1 as a common cargo receptor that binds Aβ/carrier complexes (Goto and Tanzi, 2002; Moir and Tanzi, 2005; Harris-White and Frautschy, 2005). Within the cells, the binding of Aβ to LRP1 allows the LRP1 to carry Aβ molecules to the basolateral inner membrane facing the blood with the subsequent expelling of Aβ into the blood stream (Deane et al., 2004; Donahue et al., 2006). LRP1 has been shown to bind both Aβ40 and Aβ42, alone, or conjugated to one of their carrier proteins, facilitating its transport (Jaeger and Pietrzik, 2008). In ageing brains, a decrease in LRP1 presence in the brain–blood barrier (BBB) has been observed (Silverberg et al., 2010), whereas, an increase in the B-CSF-B was described (Pascale et al., 2011). In our ChP organoid system, LRP1 expression levels increased in the presence of Aβ. Our findings agree with previous studies where intracellular and soluble forms of LRP1 are increased in ChP and CSF with advancing age (Jaeger and Pietrzik, 2008; Liu et al., 2009). This suggest that the model closely mimics amyloid uptake in the ChP, which is something that has been observed in AD pathology, although more mechanistic studies using inhibitors are necessary to confirm amyloid uptake is mediated by LRP1.

Of particular interest is the fact that ChP organoids secrete a CSF-like fluid containing proteins and other known biomarkers relevant to the study and diagnosis of AD, such as the Aβ42/40 ratio (Pellegrini et al., 2020; Lewczuk et al., 2017). To test the functional role of ChP organoids as a selective barrier, we examined the entry of fluorescently labelled Aβ42 into CSF-like fluid secreted by the organoids. The results showed that the barrier allows the passage of Aβ42, as well as the secretion of Aβ40. Since LRP1 expressed in B-CSF-B facilitates the expulsion of Aβ into the blood stream (Deane et al., 2004; Donahue et al., 2006), and that LRP1 is indeed expressed in our ChP organoid model, our findings corroborate the *in vivo* previously published data, supporting this model for studying *in vivo* Aβ uptake.

There is growing pressure on researchers to reduce their use of animals in experiments. Organoids are miniature *in vitro* models of human organs that are characterized by their similarity in its architecture and physiology to organs (Sainz et al., 2024). Particularly, brain organoids offer several advantages for studying neurodegeneration by providing a more accurate, human-relevant 3D model than traditional 2D systems or animal models. They can replicate key brain structures and cell types, enabling researchers to study disease mechanisms, test new therapies, and develop personalized treatments using patient-derived cells. Here, we present new evidence of the ability of ChP organoids to mimic ChP activity/function, and to replicate physiological tissue organization. In recent years, brain organoid models have significantly advanced our understanding of the progression of neurodegeneration, particularly in AD (Zhao et al., 2020). Increasing the *in vitro* culturing time of organoids from weeks to months produces CSF-like fluid that that closely resembles native human CSF. ChP organoids allows for a detailed study of the Aβ uptake and crossing through the B-CSF-B, which has not been possible until now.

### 4.1 Limitations and future directions

Although ChP organoids provide a promising human in vitro model, they do not yet capture ageing-associated signatures that are highly relevant in AD (Pellegrini and Lancaster, 2021; Faravelli et al., 2020). In this proof of principle study, sample sizes were modest and therefore statistical power is limited; however, key findings were validated using orthogonal approaches. We also did not perform direct barrier permeability or tightness assays or include a comparison with human primary ChP tissue.
Notably, while aging is the primary risk factor for AD, gradual molecular, structural and functional changes begin to occur decades before the onset of clinical symptoms. Future work, using chronic exposure to amyloidogenic peptides or other forms of stress such as hypoxia or chronic injury, may help to induce features more representative of the disease’s late-onset nature.

## 5 Conclusion

Taken together, this model provides a platform to address significant knowledge gaps and offers a novel approach to investigating the development and progression of AD. By recreating essential features of the human ChP biology in a tractable system, it enables mechanistic investigation of how the ChP and the B-CSF-B are altered during disease onset and progression. We anticipate that this model will help dissect pathogenic pathways and may support the development of therapeutic interventions for AD.

## Data Availability

The raw data supporting the conclusions of this article will be made available by the authors, without undue reservation.
