# Supplementary material for: Choroid plexus organoids mimic amyloid uptake at the blood-cerebrospinal fluid-barrier
Source: Front Cell Neurosci. 2026 Feb 18;20:1769911. doi: 10.3389/fncel.2026.1769911 (PMC12956682; doi:10.3389/fncel.2026.1769911)
Supplement: Supplementary file 1 [file Table_1.DOCX]

Supplementary Table. Organoids batches used for CSF collection.

| **Cell Line** | **Batch n.** | **Days in vitro** | **ChP diff** | **volume** | **notes** |
| --- | --- | --- | --- | --- | --- |
| **H1** | 1 | 46 | no | >25ul | untreated organoids that spontaneosly developed ChP |
| **H1** | 2 | 48 | yes | ±25ul |  |
| **H1** | 3 | 102 | yes | >25ul |  |
| **H1** | 4 | 131 | yes | >25ul |  |
| **H1** | 5 | 132 | yes | >25ul |  |
| **H9** | 6 | 60 | no | >25ul | untreated organoids with both cortical tissue and ChP |
| **H1 Aβ1** | 7 | 42 | yes | >25ul | organoids treated with Aβ seeds 48h |
